# Study of FOXO1/pFOXO1, lncRNA ADAMTS9-AS2, and miR-96-5p in laryngeal squamous cell carcinoma

**DOI:** 10.1186/s12885-025-15283-6

**Published:** 2025-12-23

**Authors:** Masoomeh Bakhshandeh, Payam Mohammadi, Najmeh Parvaz, Maryam Lotfi, Omid Joodi, Mohammad Najafi

**Affiliations:** 1 Clinical Biochemistry Department, Faculty of Medical Sciences, Iran University of Medical Sciences, Tehran, Iran; 2 Otorhinolaryngology Research Center, Amir Alam Hospital, Tehran University of Medical Sciences, Tehran, Iran

**Keywords:** LSCC, FOXO1, ADAMTS9-AS2, MiR-96-5p

## Abstract

**Background:**

Laryngeal squamous cell carcinoma (LSCC) is recognized as the second most common malignant tumor of the respiratory tract. The study aimed to identify the roles of FOXO1, hsa-miR-96-5p, and lncRNA ADAMTS9-AS2 in the molecular pathogenesis of LSCC patients based on the systems biology data.

**Methods:**

The LSCC patient tissue samples (*n* = 50) and the same individual’s adjacent normal tissues (*n* = 50) were collected from the candidates (aged 57.75 ± 9.3 years) of surgery. The miR-96-5p and lncRNA ADAMTS9-AS2 were predicted using the specific servers. The Kaplan Meier analysis was employed using TCGA data. The FOXO1and ncRNA gene expression levels were measured with the RT-qPCR technique. The Western blot technique was applied to estimate FOXO1/pFOXO1 protein values.

**Results:**

A FOXO1/miR-96-5p/ADAMTS9-AS2 gene network was constructed and enriched using the bioinformatics data. The FOXO1 (p 0.037) correlated with ADAMTS9-AS2 (p 0.04) gene expression levels and was reduced in the LSCC patient tissue samples despite the elevated miR-96-5p expression levels (p 0.047). Moreover, the FOXO1 (*p* < 0.01) and pFOXO1 (*p* < 0.0001) protein values were reduced in the LSCC. The high FOXO1 and ADAMTS9-AS2 gene expression levels significantly increased the survival probability (HR 0.61 and 0.65, respectively).

**Conclusion:**

The FOXO1 and ADAMTS9-AS2 genes might act as molecular suppressors in the cell growth pathways. Furthermore, miR-96-5p is suggested as an oncogenic miRNA in the LSCC.

**Supplementary Information:**

The online version contains supplementary material available at 10.1186/s12885-025-15283-6.

## Introduction

Laryngeal squamous cell carcinoma (LSCC), the principal malignancy in otolaryngology, is known as the second most common primary malignant tumor in the respiratory tract [1]. The studies showed that LSCC incidence is significantly higher in males than in females [2]. Among risk factors reported including smoking, alcohol, exposure to air pollution, industrial chemicals, infections such as Human papillomavirus (HPV), food habits, clinical history like gastroesophageal reflux disease (GERD) and laryngopharyngeal reflux (LPR), genetics is reported as an important molecular pathologic agent in the LSCC [2, 3] so understanding the cellular and molecular signaling pathways may elucidate biological targets for innovative therapeutic strategies [4].

The studies reported that the PI3K/AKT signaling pathway is involved in cancer development and progression. The Forkhead box O (FOXO) family is recognized as a key effector of the PI3K/AKT signaling pathway. FOXO1 is an important target of AKT, consistent with its role as a tumor suppressor [5]. It is involved in survival, proliferation, differentiation, and tumorigenesis processes via cross-talks with other cellular pathways such as the AMPK signaling pathway (hsa04152), Longevity regulating pathway (hsa04211), Cellular senescence (hsa04218), Insulin signaling pathway (hsa04910), Pathways in cancer (hsa05200), and Transcriptional misregulation in cancer (hsa05202). Furthermore, FOXO1 is reported to prevent tumorigenesis and is dysregulated in many cancers, such as prostate, breast, and cervical cancer [6–8].

In addition to the role of genes, other regulators such as long noncoding RNAs (lncRNAs), circular RNAs, and microRNAs (miRNAs) control cellular biological processes. There is mounting evidence that non-coding RNAs (ncRNAs), as oncogenes or suppressors, can affect epigenetics, transcriptional, and post-transcriptional events during tumor growth [9–12]. Many studies reported the roles of ncRNAs in the progression and development of LSCC. One study investigated the oncogenic role of circMYLK via the miR-195/cyclin D1 axis in LSCC progression [13]. ST7-AS1, as an oncogenic lncRNA, was also reported through the CARM1/Sox-2 axis [14]. The hsa_circ_0042666 was suggested to regulate the proliferation of LSCC cells via the miR-223/TGFBR3 axis [15]. In this category, high-throughput techniques such as RNAseq and scRNA have been extensively investigated to study the changes of ncRNAs and genes [16–20].

These reports indicated that further studies are necessary to identify and clarify the cellular signaling axes and pathways involved in the LSCC pathogenesis. Therefore, this study was designed to examine the changes in FOXO1/pFOXO1, hsa-miR-96-5p, and lncRNA ADAMTS9-AS2 in LSCC and their impact on the survival probability of TCGA patients based on systems biology data.

## Methods

### Sample

**Fig. 1:**
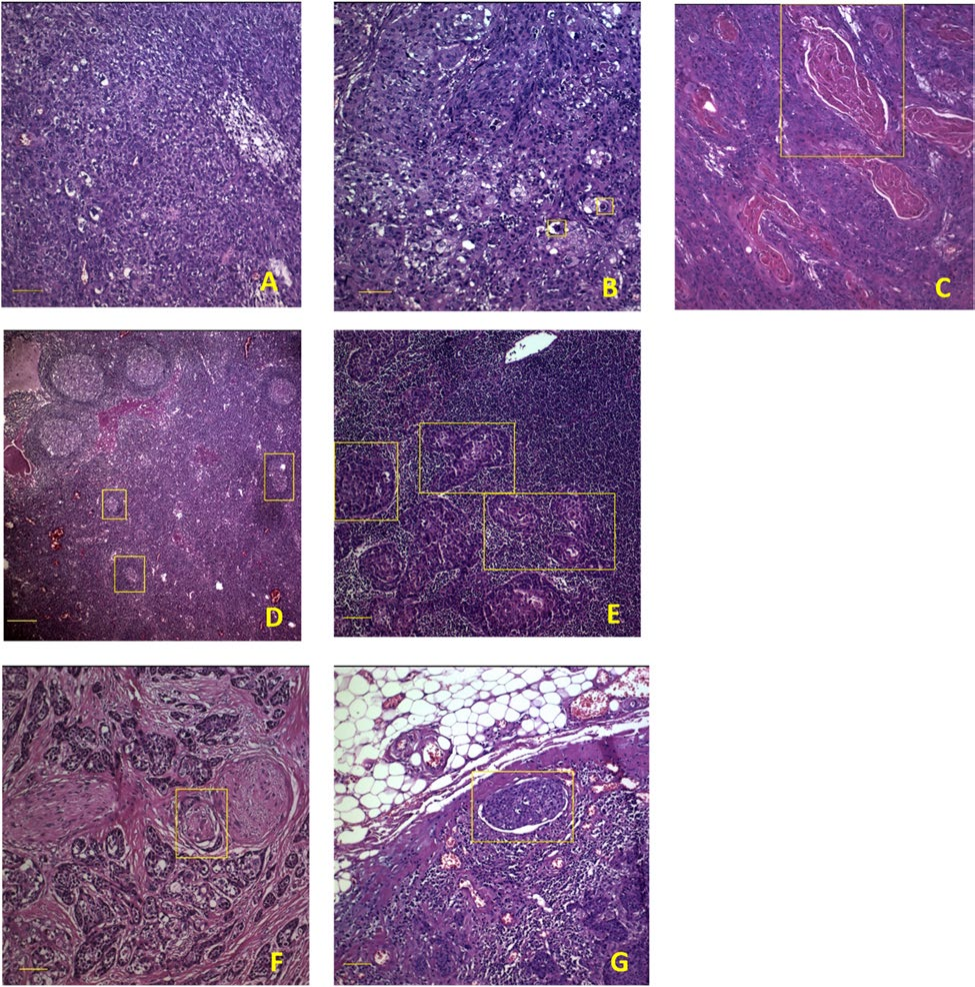
Pathological images. **A**, Poorly differentiated (Scale bar: 0.1 mm, magnification: 10x). **B**, Moderately differentiated (Scale bar: 0.1 mm, Magnification: 10x, Yellow rectangle: keratin pearl). **C**, Well differentiated (Magnification: 10x, Yellow rectangle: keratin pearl). **D**, Free or normal lymph node (Scale bar: 0.025 mm, Magnification: 4x, Yellow rectangle: germinal center). **E**, Involved lymph node (Scale bar: 0.1 mm, Magnification: 10x, Yellow rectangle: tumor cell). **F** Perineural invasion (Scale bar: 0.1 mm, Magnification: 10x, Yellow rectangle: PNI). **G** Lymphovascular invasion (Scale bar: 0.1 mm, Magnification: 10x, Yellow rectangle: LVI)

Fifty tissue samples of laryngeal squamous cell carcinoma (LSCC) were collected from male patients who were candidates for surgery and had been enrolled exclusively in the Tumor Bank of the Otorhinolaryngology Research Center at Amir A’lam Hospital, Tehran, Iran (Table 1). The same individual’s adjusted healthy tissues (their adjacent normal tissues, *n* = 50) were applied as control samples. A postoperative pathological examination was performed according to AJCC’s criteria for all the cases (Table 2). The cellular features, such as nuclear shape, cell arrangement, and abnormal growth patterns, were examined in the stained tissue samples using hematoxylin and eosin (H&E). The distinct markers linked to malignancy and differentiation, like irregular cell clusters or keratinization, were identified (Fig. 1).

**Table 1:** Demographic factors in the study population

Parameter	Value Mean ± SD %
Age (Year)	57.75 ± 9.3
Smoking (No/Yes)	20.8/79.2
Alcohol (No/Yes)	93.1/6.9
Narcotics (No/Yes)	61.1/38.9

**Table 2:** Pathological characteristics of the LSCC tissue samples

Parameter	Case %
Nodes involvement ^a^ (No/Yes)	68.6/31.4
Vocal cord involvement ^b^ (No/Yes)	37.5/62.5
Thyroid cartilage involvement ^c^ (No/Yes)	40.3/59.7
Extra-laryngeal soft tissue invasion ^d^ (No/Yes)	49.7/40.3
Epiglottis invasion ^e^ (No/Yes)	38.9/61.1
Peri-neural invasion ^f^ (No/Yes)	68.1/31.9
Lymphovascular invasion ^g^ (No/Yes)	72.2/27.8
Stage ^h^ (T3/T4)	40.3/59.7
Histological differentiation ^i^ (Poor or moderate/Well)	33.3/65.3

^a^ Node involvement: Defined based on pathological examination of cervical lymph nodes; "No" indicates absence of metastatic lymph nodes, "Yes" indicates presence of metastasis to at least one lymph node (based on AJCC 8th edition criteria)

^b^ Vocal cord involvement: Determined via direct laryngoscopy and imaging; "Yes" indicates tumor extension to one or both true vocal cords, regardless of mobility

^c^Thyroid cartilage involvement: Defined as invasion into or through the thyroid cartilage as assessed by radiologic imaging (CT/MRI) or intraoperative findings

^d^ Extra-laryngeal soft tissue invasion: Tumor extension beyond the larynx into surrounding soft tissue spaces (AJCC)

^e^ Epiglottis invasion: Defined as tumor infiltration into the suprahyoid or infrahyoid epiglottis, determined via imaging and/or endoscopy

^f^ Peri-neural invasion (PNI): Presence of cancer cells tracking along or around a nerve, identified in histopathological examination

^g^ Lymphovascular invasion (LVI): Presence of tumor cells within lymphatic and/or vascular channels, detected on H&E staining of tumor tissue

^h^ Stage: Based on tumor (T) stage (AJCC); T3 indicates limited laryngeal fixation or deep invasion without cartilage destruction; T4 indicates moderately advanced or very advanced local disease

^i^ Histological differentiation: Graded according to WHO criteria; "Well-differentiated" tumors show keratinization and mature squamous features; "Poorly or moderately differentiated" tumors have less organization and more cellular atypia

### miRNA/lncRNA prediction

The miRNAs and lncRNAs were predicted based on their associations with FOXO1 in databases. A gene/miRNA/lncRNA network was constructed and enriched on the high-score edges. The mirtarbase [21], Diana [22, 23], mirdb [24], miRmap [25], mirwalk [26], Starbase [27], Targetscan [28], TCGA (https://www.cancer.gov/ccg/research/genome-sequencing/tcga), and GEO (including GSE133632 and GSE142083) were used to predict the miRNA. First, a total summed score was estimated through the reports of miRNAs in databases to associate with the FOXO1 gene (Supplementary 1). Then, the hsa-miR-96-5p was selected through high-score miRNAs with no reports in the LSCC. The lncRNA ADAMTS9-AS2 was also predicted to associate with hsa-miR-96-5p expression changes through total scores estimated for Starbase (Encori), RNAinter [29], Diana [30], TCGA, and GEO (including GSE117007 and GSE142083) [31] databases (Supplementary 2). To prioritize ncRNAs based on evidence strength, a scoring system was developed that integrates both predictive and experimental data. Prediction-based entries from the above databases were assigned a score of 0.5, while experimentally validated ncRNAs from TCGA and GEO datasets received a score of 1.0. To further emphasize biological relevance, the experimental score was multiplied by the Log Fold Change (LogFC), amplifying the impact of ncRNAs with significant expression changes.

$$\:\mathrm{Total}\:\mathrm{Score}=\mathrm n\times\:0.5+\left(\mathrm{LogFC}\:\left(\mathrm{TCGA}\right)+\mathrm{LogFC}\:\left(\mathrm{GEO}\right)\right)\times\:1$$

### RNA extraction and cDNA synthesis

The total RNA was extracted using the BIO Basic total RNA extraction kit (BIO BASIC CANADA Inc., Canada) according to the producer’s instructions. The RNA quality in each sample was determined by its optical absorbance ratio at 260/280 nm using a NanoDrop One (Thermo Fisher Scientific, Waltham, Massachusetts, United States). cDNA was synthesized using the cDNA synthesis kit (Zist Virayesh, Tehran, Iran) using nonamers/hexamers for the genes and a stem loop for the miRNA. The RNA and cDNA samples were preserved at −80 °C.

### Gene expression measurement

Specific primers for hsa-miR-96-5p and lncRNA ADAMTS9-AS2 were designed using Primer-BLAST (NCBI, https://www.ncbi.nlm.nih.gov/tools/primer-blast) and checked with the Oligo-Analyzer server (Integrated DNA Technologies, https://www.idtdna.com/pages/tools/oligoanalyzer) (Table 3). The FOXO1, hsa-miR-96-5p, and ADAMTS9-AS2 expression levels were measured with the RT-qPCR technique (Zist Virayesh high ROX SYBR Green qPCR Master Mix kit, Tehran, Iran) using StepOne Real-time PCR System (Thermo Fisher Scientific, Waltham, Massachusetts, United States). The GAPDH and U6 expression levels were used to normalize the FOXO1, ADAMTS9-AS2, and hsa-miR-96-5p expression levels.

**Table 3:** Primers

Gene	Sequence (5’→ 3’)
FOXO1	F: TACGAGTGGATGGTCAAGAGCGTG
	R: CCTGCCACCCTCTGGATTGAGCA
ADAMTS9-AS2	F: TAAGACCCACGAACGACAGCG
	R: GCTTTCAGCCAGACATCAGGGTTT
hsa-miR-96-5p	F: GGGAGGGTTTGGCACTAGCACATT
	R: CGTTGGCTCTGGTGCTGGGT
	Loop: CGTTGGCTCTGGTGCTGGGTCCGAGGTATTCGCACCAGAGCCAACGAGCAAA
GAPDH	F: CATGAGAAGTATGACAACAGCC
	R: AGTCCTTCCACGATACCAAAGT
U6	F: TTGGAACGATACGGAGAAGATTAGC
	R: TATGGAACGCCTCACGAATTTGC

### FOXO1/pFOXO1 protein measurement

The FOXO1/pFOXO1 protein values were measured using the modified Western blot technique [32, 33]. The total protein was extracted with RIPA buffer after thoroughly crushing the tissue with a homogenizer, and was determined with the Protein Quantification kit (DB0017, DNAbioTech, Iran). The lysate was collected through centrifugation (14,000 rpm, 20 min, 4 °C) and was mixed with an equal volume of 2x Laemmli sample buffer. After boiling (5 min), the protein sample (20 µg) was electrophoresed on a sodium dodecyl sulfate-polyacrylamide (SDS-PAGE) gel and then transferred to a 0.2 μm immune-Blot™ polyvinylidene difluoride (PVDF) membrane (Cat No: 162–017777; Bio-Rad Laboratories, CA, USA). The membrane was blocked with 5% BSA (Cat No: A-7888; Sigma Aldrich, MO, USA) in 0.1% Tween 20 (one hour). After washing with PBS, it was incubated with anti-FOXO1 (1/1000, Cat No: 9454 S; Cell Signaling) and anti-pFOXO1 (Ser256) (1/1000, Cat No: 84192 T; Cell Signaling), as well as Anti-GAPDH-loading control antibodies (1/2500, Cat No: ab8245, Abcam) (one hour, room temperature). The membrane was then washed three times with TBST and incubated with goat anti-rabbit IgG H&L (HRP) (1/10000, Cat No: ab6721; Abcam) secondary antibodies. Afterward, it was exposed to enhanced chemiluminescence (ECL) reagent (1–2 min). The protein band densitometry analysis was performed using ImageJ software (1.52v). The protein expression values were normalized to the GAPDH values (Supplementary 3).

### Statistical analysis

The data were analyzed using SPSS Statistics software (Ver. 27.0, SPSS Inc., Chicago, IL) and GraphPad Prism (Ver. 10.4.1) software. Cytoscape software (Ver. 3.8.1) was used to visualize the gene network. The data distribution was identified using the Kolmogorov-Smirnov test. The differences between groups were evaluated using the Mann-Whitney and Student’s t tests. The data correlations were estimated by the Spearman test. Kaplan Meier analyses were employed to identify the survival probabilities among Head and Neck squamous cell carcinoma patients (TCGA, Gender: male, and Stage: 3 and 4) using KM Plotter [34]. The Log-rank *P* values and Hazard Ratios (95% CI) were estimated to evaluate the low and high FOXO1, hsa-miR-96-5p, and ADAMTS9-AS2 expression changes with the risk of mortality. The *p* value < 0.05 was considered significant throughout the analysis process. The relative gene expression levels were calculated by the 2^−ΔΔCT^ formula.

## Results

### Prediction of gene/miRNA/lncRNA network

**Fig. 2:**
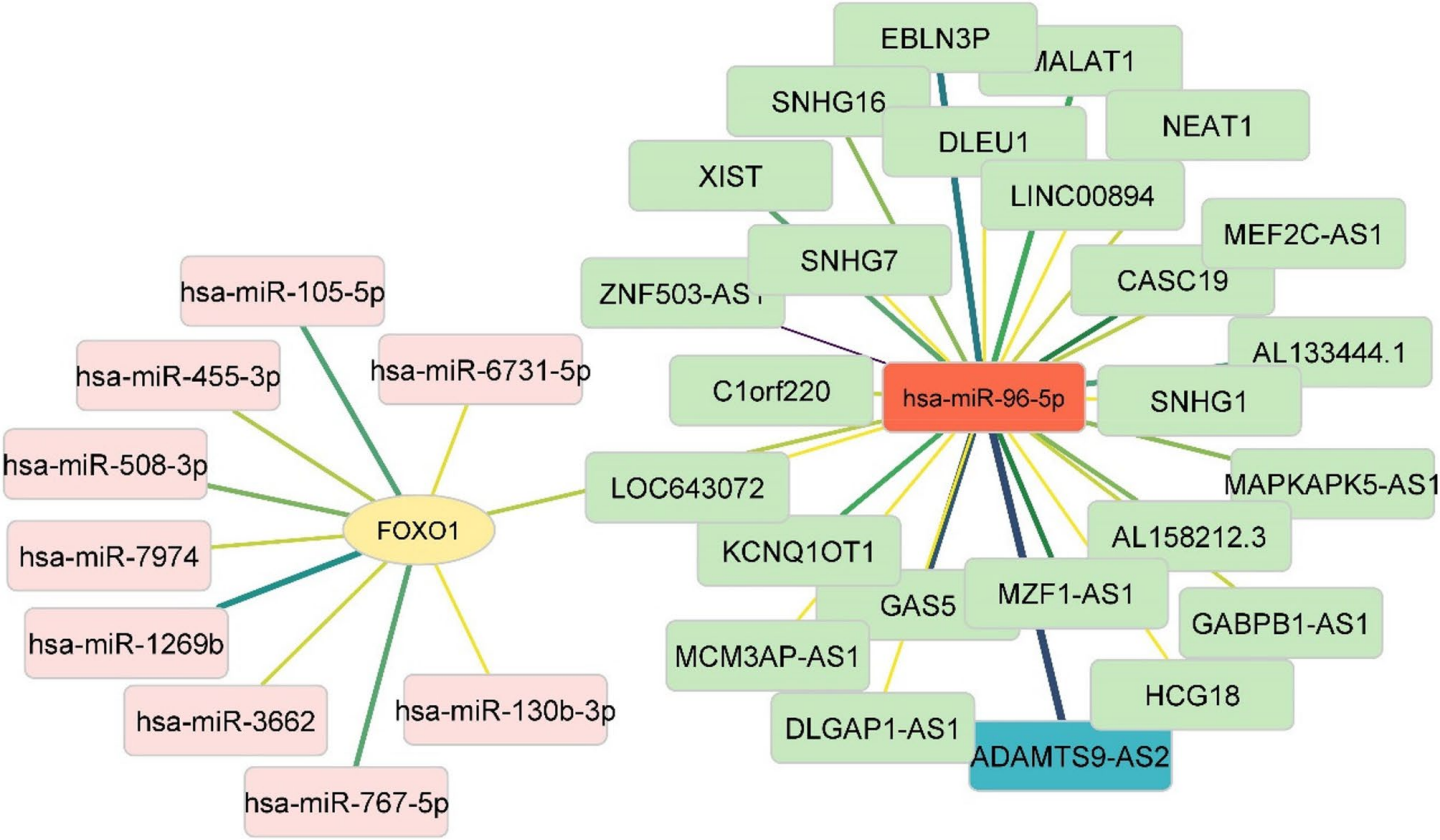
Gene/miRNA/lncRNA network. The associations between high-score miRNAs (pink), FOXO1 gene (yellow), and high-score lncRNAs (green) are shown on the network. The hsa-miR-96-5p (red) and ADAMTS9-AS2 (blue) were selected in this study. Yellow edge, the lowest score; Black edge, the highest score

A gene/miRNA/lncRNA network was constructed according to the ncRNAs scores (Fig. 2). The hsa-miR-96-5p had the most database reports, so it was selected in this study through high-score miRNAs (Top10) on the network. Moreover, the high-score lncRNAs associated with the hsa-miR-96-5p were predicted and presented on the network (*n* = 25). The lncRNA ADAMTS9-AS2 (highest score 8.019) was selected for the study (Fig. 2).

### The FOXO1, hsa-miR-96-5p, and ADAMTS9-AS2 expression levels

**Fig. 3:**
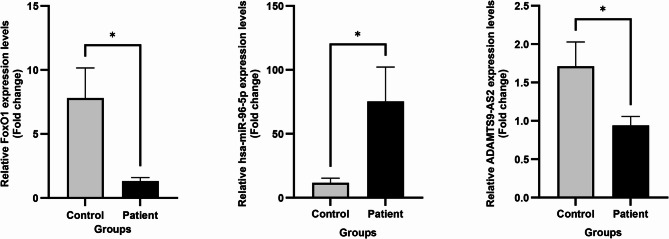
FOXO1, hsa-miR-96-5p, and ADAMTS9-AS2 gene expression levels between the LSCC patient and control tissue samples. Mean ± SEM, * *P*-Value < 0.05

The FOXO1, hsa-miR-96-5p, and ADAMTS9-AS2 gene expression levels revealed significant differences between the LSCC patient and control tissue samples (Fig. 3). In contrast with the hsa-miR-96-5p expression levels (p 0.047), the FOXO1 and ADAMTS9-AS2 gene expression levels were reduced in the LSCC patient samples (p 0.037 and 0.04, respectively).

### The FOXO1/pFOXO1 protein expression levels

**Fig. 4:**
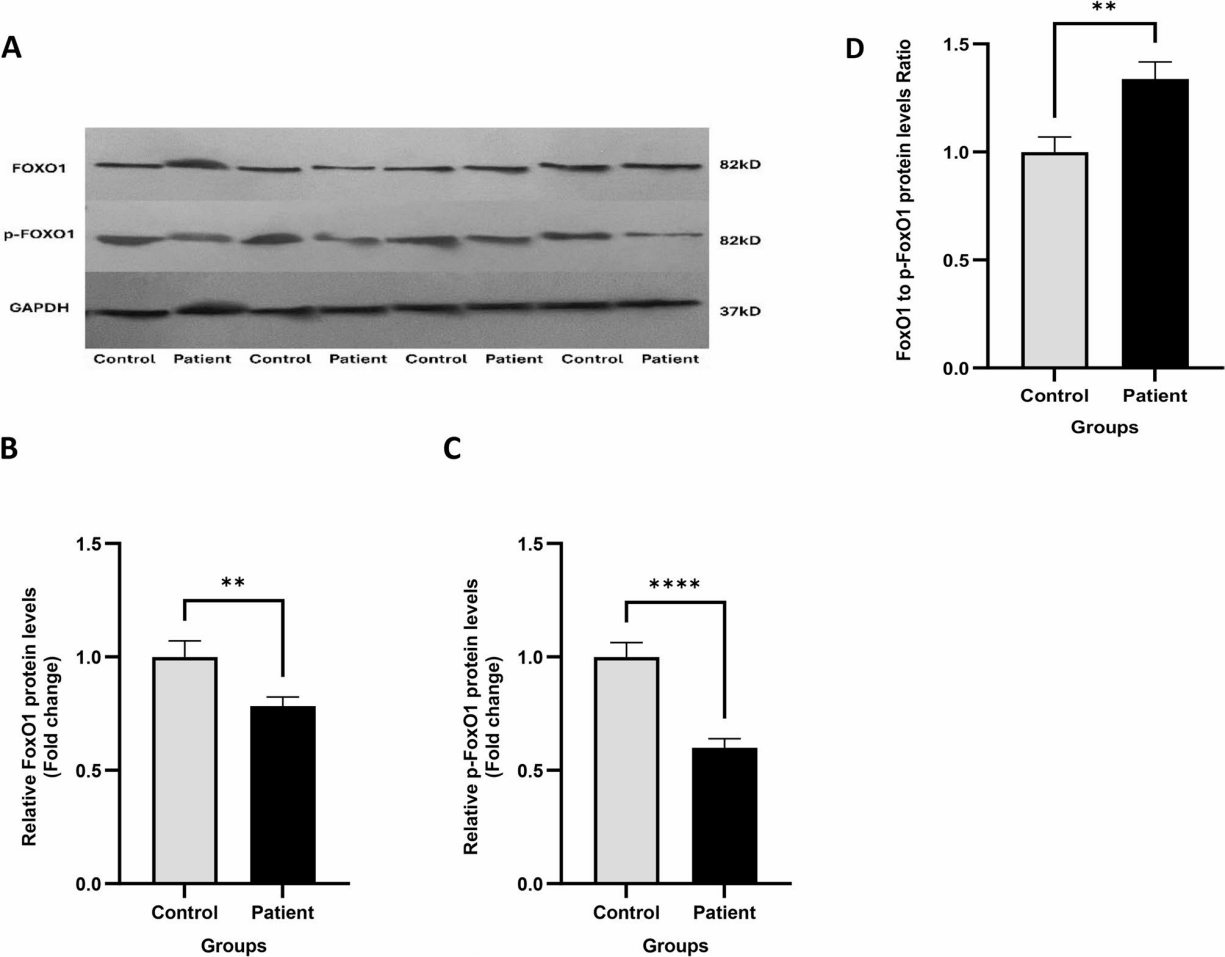
The FOXO1 and pFOXO1 protein expression levels. **A**, Western blot image. **B**, FOXO1. **C**, pFOXO1. **D**, FOXO1/pFOXO1 protein ratio. Mean±SEM. **,*P* < 0.01. **** *P* < 0.0001

The FOXO1 and pFOXO1 protein expression levels were reduced significantly between the LSCC patient and control tissue samples (*p* < 0.01) (Fig. 4). The FOXO1/pFOXO1 ratio increased significantly in LSCC tissues as compared to control samples (*p* < 0.01).

### FOXO1, ADAMTS9-AS2 and miRNA correlations

Correlations between the protein and gene data were provided after internalizing the effects of Chemo/Radiotherapy, smoking, alcohol, and narcotics confounders. The results showed significant correlations between FOXO1 gene expression levels, lncRNA ADAMTS9-AS2 (p 0.002), and protein expression levels (p 0.049) as shown in Table 4.

**Table 4:** Correlations between FOXO1/pFOXO1, ADAMTS9-AS2 and hsa-miR-96-5p in the study population

Parameter	FOXO1 gene Correlation Coefficient, (*P*-value)	ADAMTS9-AS2 Correlation Coefficient, (*P*-value)	hsa-miR-96-5p Correlation Coefficient, (*P*-value)
FOXO1 gene	-	0.358, (0.002)	0.129, (0.283)
lncRNA ADAMTS9-AS2	0.358, (0.002)	-	−0.112, (0.353)
hsa-miR-96-5p	0.129, (0.283)	−0.112, (0.353)	-
FOXO1 protein	0.397, (0.049)	−0.117, (0.576)	−0.231, (0.268)
pFOXO1 protein	0.186, (0.373)	−0.315, (0.126)	−0.117, (0.397)

### FOXO1, ADAMTS9-AS2, miR-96-5p and pathological characteristic relationships

The relationships between the gene and protein expression levels and the pathological features were evaluated in LSCC patient tissue samples (Table 5). The miR-96-5p expression was significantly associated with lymph node involvement (*P* = 0.039). Patients without lymph node metastasis exhibited a lower miR-96-5p expression level. In contrast, patients with lymph node involvement showed a higher miR-96-5p expression level. FOXO1 protein levels were significantly lower in LSCC patients without perineural invasion (*P* = 0.024), as shown in Table 5.

**Table 5:** Relationships between the gene and protein expression levels and the clinicopathological features of LSCC patient tissue samples

Clinicopathological features		Gene Expression						Protein Expression					
		FOXO1		ADAMTS9-AS2		hsa-miR-96-5p		FOXO1 protein		pFoxO1 protein		FOXO1/pFOXO1 ratio	
		Range (ΔCt _min−max_)	*P*	Range (ΔCt _min−max_)	*P*	Range (ΔCt _min−max_)	*P*	Relative Intensity	*P*	Relative Intensity	*P*	Relative Intensity	*P*
Nodes involved	N0	2.48–14.03	0.470	5.42–13.20	0.394	3.47–21.09	0.039	0.41–1.14	0.722	0.53–1.69	0.509	0.46–1.45	0.875
	>N0*	5.87–18.20		7.13–17.71		0.62–15.29		0.41–1.09		0.58–1.55		0.53–1.24	
Vocal cord involvement	No	7.27–18.20	0.436	8.37–17.71	0.013	0.62–13.16	0.916	0.41–1.09	0.450	0.58–1.42	0.612	0.51–1.33	0.404
	Yes	5.87–16.94		7.13–12.57		1.04–15.29		0.50–1.45		0.53–1.69		0.46–1.45	
Thyroid cartilage involvement	No	6.32–15.02	0.649	7.13–15.14	0.745	0.62–15.29	0.168	0.41–1.14	0.179	0.58–1.48	0.884	0.51–1.05	0.101
	Yes	5.87–18.20		7.29–17.71		1.04–14.26		0.50–1.09		0.53–1.69		0.46–1.45	
Extralaryngeal soft tissue invasion	No	6.32–16.94	0.540	7.13–15.14	0.500	0.62–15.29	0.918	0.41–1.14	0.059	0.53–1.69	0.905	0.46–1.45	0.126
	Yes	5.87–18.20		7.29–17.71		1.34–14.26		0.56–1.09		0.59–1.55		0.53–1.33	
Epiglottis invasion	No	5.87–13.57	0.759	7.13–12.93	0.922	0.62–14.98	0.126	0.50–0.85	0.076	0.53–1.69	0.891	0.46–1.05	0.157
	Yes	6.32–18.20		7.52–17.71		1.70–15.29.70.29		0.41–1.14		0.58–1.55		0.51–1.45	
Perineural invasion	No	5.87–15.02	0.924	7.13–17.71	0.310	1.04–15.29	0.321	0.50–1.14	0.024	0.53–1.69	0.082	0.46–1.33	0.886
	Yes	7.27–18.20		8.01–15.14		0.62–14.26		0.41–0.88		0.58–1.55		0.51–1.45	
Lymphovascular invasion	No	5.87–18.20	0.456	7.52–15.14	0.513	0.62–15.29	0.233	0.41–1.14	0.689	0.53–1.48	0.818	0.51–1.45	0.816
	Yes	7.61–14.64		7.13–17.71		1.04–14.26		0.41–1.09		0.58–1.69		0.46–1.33	
Stage (T3/T4)	T3	6.32–15.02	0.649	7.13–15.14	0.745	0.62–15.24	0.168	0.41–1.14	0.179	0.58–1.48	0.884	0.51–1.05	0.101
	T4	5.87–18.20		7.29–17.71		1.04–14.26		0.50–1.09		0.53–1.69		0.46–1.45	
Histological differentiation ^**^	M	6.66–14.64	0.840	7.13–15.14	0.591	1.04–14.98	0.773	0.41–1.09	0.062	0.53–1.64	0.090	0.46–1.33	0.684
	W	5.87–18.20		7.29–17.71		0.62–15.29		0.41–1.14		0.58–1.55		0.52–1.45	

*, including N1, N2 and N3. **, *M* Moderately differentiated, *W *Well differentiated

### Survival probability

**Fig. 5:**
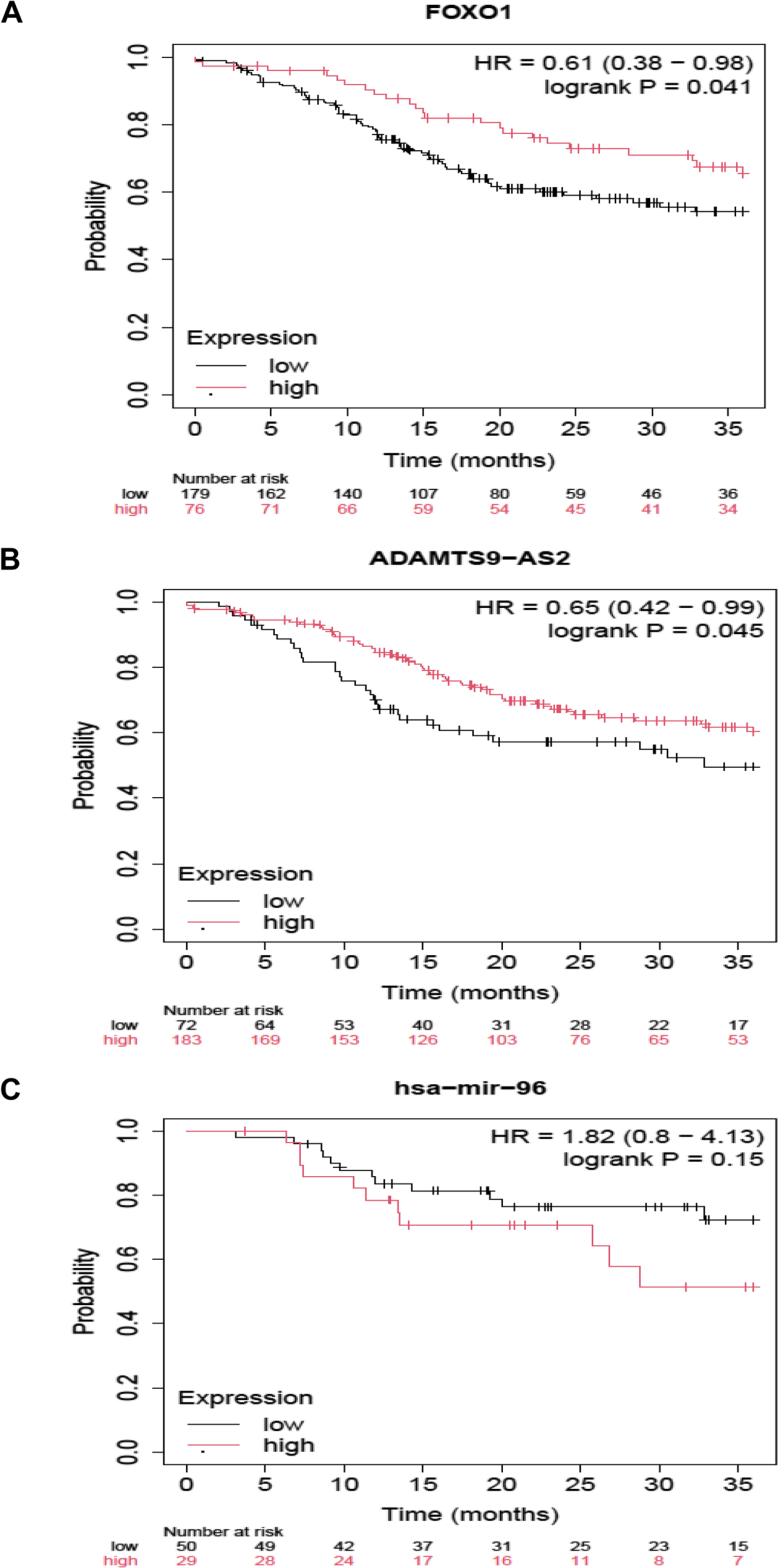
Kaplan Meier plots. **A**, FOXO1. **B**, ADAMTS9-AS2. **C**, hsa-miR-96. Survival probability is estimated among TCGA-Head and Neck squamous cell carcinoma patients with Low (Black) and High (Red) gene expression levels. HR, Hazard Ratio

The results showed that the high FOXO1 expression levels significantly elevated the survival probability among head and Neck squamous cell carcinoma patients (*n* = 255) (HR, 0.61 (0.38–0.98), log-rank *P* = 0.041), reducing the death risk up to 39% over three year period (Fig. 5A). Similar to FOXO1, the high ADAMTS9-AS2 expression levels also significantly reduced the risk of mortality up to 35% among the patients (HR, 0.65 (0.42–0.99), log-rank *P* = 0.045) (Fig. 5B). In contrast, the high miR-96 expression level increased the risk of death among the patients (*n* = 79), but it was not significant (HR, 1.82 (0.8–4.13), log-rank *P* = 0.15) (Fig. 5C).

## Discussion

**Fig. 6:**
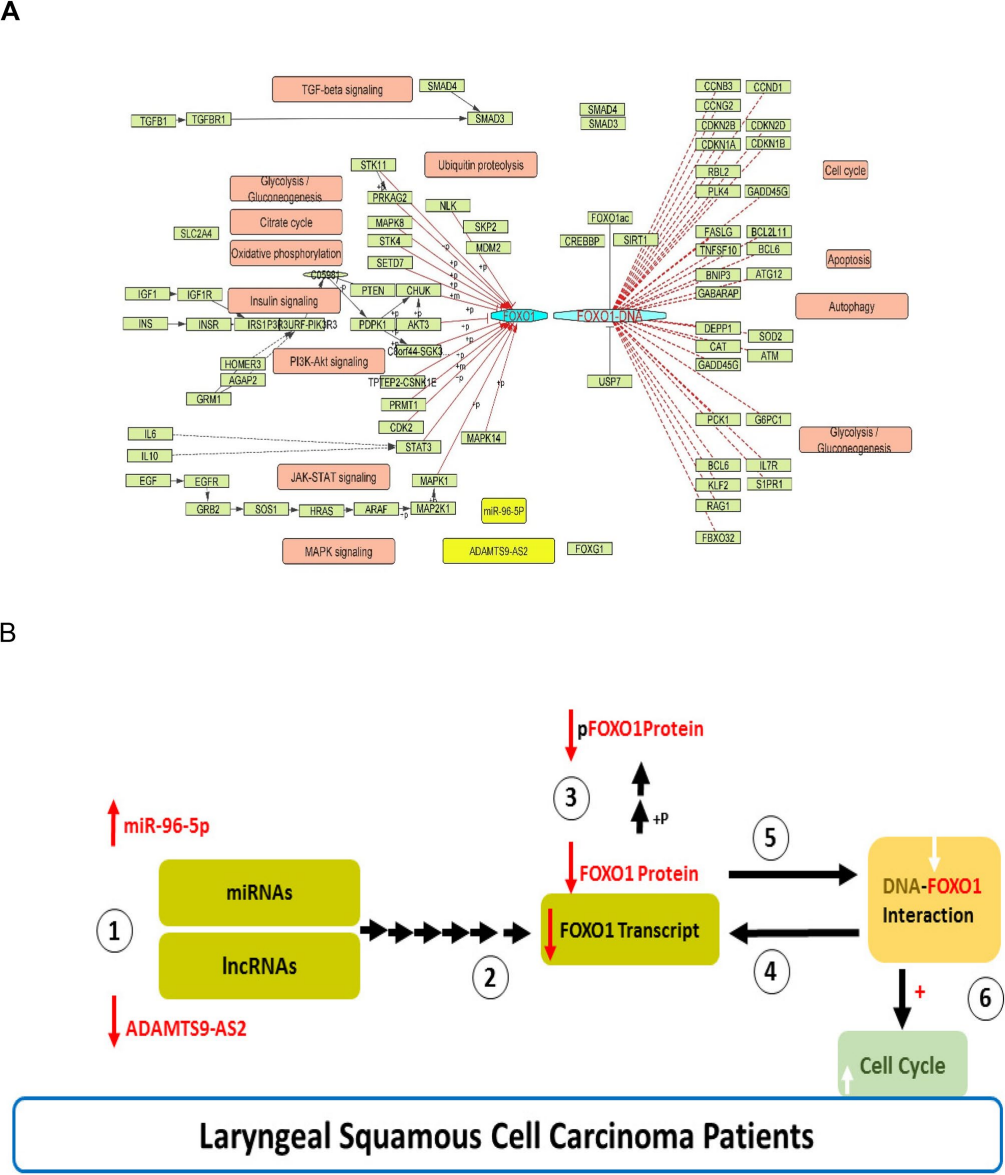
Relationships among FOXO1, miR-96-5p, ADAMTS9-AS2, and genes in diverse Signaling Pathways. **A**, Crosstalk between FOXO1 signaling pathway (hsa04068) and other cellular signaling Pathways. FOXO1 is tightly regulated by upstream cytosolic pathways, including PI3K-AKT, TGF-β, MAPK, Insulin, and JAK-STAT signaling. It also governs downstream signaling pathways involved in cell cycle progression, apoptosis, and metabolic regulation. **B**, Suggested Interplay of FOXO1, miR-96-5p, and ADAMTS9-AS2. (1) lncRNA ADAMTS9-AS2, also known as a gene suppressor, might inversely affect the miR-96-5p transcripts via a sponging mechanism or control of other genes and miRNAs involved in the miR-96-5p expression levels. (2) The increased miR-96-5p reduced the number of FOXO1 transcripts via degrading mRNA of FOXO1 or TFs involved in FOXO1 expression. (3) The reduced FOXO1 gene expression levels decreased the total FOXO1 and pFOXO1 protein values. (4) The FOXO1 expression levels also could be related to its promoter function. (5) The reduced FOXO1 protein, known as a transcriptional suppressive factor, decreased its interaction with DNA, resulting in (6) the increased cell cycle in the LSCC. Cellular studies using Luciferase reporter or RNA pull-down should validate the suggested mechanisms

Laryngeal squamous cell carcinoma (LSCC) is known as a principal malignancy in the respiratory tract. The LSCC etiology relates to some demographic factors. Moreover, many studies reported that genetics is involved in the LSCC [2, 3], so that the study of cellular and molecular signaling axes may improve molecular pathogenesis. The FOXO1 is known as an essential transcriptional suppressive factor and is involved in some cellular signaling pathways. It is regulated by ncRNAs and post-translational factors. Following phosphorylation by Akt, through the PI3K signaling pathway, the pFOXO1 is degraded, so that the activity of FOXO1/pFOXO1 axis affects cellular functions [35, 36]. Moreover, the dynamic interplay between FOXO1 and the PI3K-AKT, TGF-β, MAPK, Insulin, and JAK-STAT signaling pathways highlights its role as a molecular hub, balancing proliferative and apoptotic signals to maintain cellular homeostasis (Fig. 6 A). Based on the role of this axis in cancer biology, FOXO1 gene and protein expression levels and its phosphorylated form were studied in the LSCC patients. Although the combined Cox regression is suggested for the Kaplan Meier analysis, the univariate Cox regression model was used to evaluate based on distinct and non-overlapped cohorts in TCGA-Head and Neck squamous cell carcinoma during three years, because the median overall survival (OS) was estimated to be less than three years among patients with stages 3 and 4 [37]. Despite the elevated FOXO1/pFOXO1 ratio observed in LSCC patient samples, both total FOXO1 and its phosphorylated form (pFOXO1) were markedly reduced, indicating a global suppression of the FOXO1/pFOXO1 regulatory axis. The increased FOXO1/pFOXO1 ratio did not signify FOXO1 activation, but rather reflected a disproportionate decline in pFOXO1 levels relative to FOXO1. Notably, a basal level of FOXO1 expression was retained in the cells [38]. Furthermore, the study data showed that the FOXO1 protein values relate inversely to the perineural invasion. As observed in other malignancies [39, 40], it was likely due to the development of proliferation and migration pathways to invade the tumor into the nervous system since the LSCC patients had the T3 and T4 stages. The results also confirmed that the survival probability decreases among patients with low FOXO1 gene expression levels.

Based on the bioinformatics data, the associations between the miR-96-5p, ADAMTS9-AS2, and FOXO1 were predicted by scoring the edges in the gene network. The study data showed that the FOXO1 gene expression levels were inversely changed with the miR-96-5p gene expression levels. Although patients with elevated miR-96 expression levels showed a trend toward reduced survival probability, the Kaplan–Meier analysis did not reach statistical significance (*P* = 0.15), so the sample size (*n* = 79) may have contributed to insufficient statistical power. Additionally, the lack of distinction between miR-96-5p and miR-96-3p isoforms in the TCGA dataset may have further obscured potential biological effects. Moreover, despite the lack of correlation between FOXO1 and miR-96-5p expression levels, miR-96-5p was related to lymph node metastasis. It is known to target the tumor suppressors such as FOXF2 and regulators of epithelial-mesenchymal transition (EMT) in other cancers [41–44], likely involved in silencing gene suppressors in the LSCC. Furthermore, the study results showed that the ADAMTS9-AS2 levels correlate with FOXO1 gene expression levels, suggesting the existence of a co-regulatory approach. It was potentially suggested in its role in sponging miRNAs, as reported for other miR-27a-3p [45]. Some studies reported that the ADAMTS9-AS2 is known as a tumor suppressor, so it inhibits the remodeling of extracellular matrix (ECM), which is a critical process for tumor invasive development [46]. Thus, the reduction of ADAMTS9-AS2 gene expression levels is suggested to be a gene suppressor in the LSCC patients. It is also involved with the vocal cord, enabling tumor localized spread into vocal cord tissues, a mechanism corroborated by its role in sequestering oncogenic miR-196b-5p in gastric and renal carcinomas [47]. The risk of mortality was also high among the patients with decreased ADAMTS9-AS2 gene expression levels, as considered on the Kaplan Meier analysis.

In conclusion, the role of the FOXO1/miR-96-5p/ADAMTS9-AS2 axis on cellular growth is suggested in the diagram of Fig. 6B. According to the study findings, the FOXO1 gene and protein expression levels were suppressed. It is proposed to reduce the FOXO1/DNA interaction, ultimately improving cellular proliferation and migration pathways in LSCC patients. Moreover, in agreement with bioinformatics data, a positive correlation was observed between FOXO1 and ADAMTS9-AS2 gene expression levels, suggesting as gene suppressors and are involved in controlling the expression of other genes or sponging onco-miRNAs. The results also showed that miR-96-5p expression levels increase in LSCC patients, suggesting an oncogenic miRNA role in tumor development. Future studies should investigate directly the binding interactions to be validated by luciferase reporter or RNA pull-down in cellular experiments. Moreover, cross-talk of the FOXO1/pFOXO1 axis with other signaling axes should be evaluated in larger cohorts.

## Supplementary Information

Supplementary Material 1: High score miRNAs.

Supplementary Material 2: High score lncRNAs.

Supplementary Material 3: Western Blot.

## Data Availability

The data supporting the findings of the study are available on request from the corresponding author.

## Abbreviations

LSCC: Laryngeal squamous cell carcinoma
FOXO1: Forkhead box protein-containing O subfamily-Type1
FOXF2: Forkhead box protein-containing F subfamily-Type2
ncRNA: Non-coding RNA
lncRNA: Long Non-coding RNA
miRNA: MicroRNA
ECM: Extracellular matrix
EMT: Epithelial-mesenchymal transition
PI3K: Phosphoinositide 3-kinases
AKT: Protein kinase B (PKB)
TF: Transcription Factor
