# Supplementary material for: Study of FOXO1/pFOXO1, lncRNA ADAMTS9-AS2, and miR-96-5p in laryngeal squamous cell carcinoma
Source: BMC Cancer. 2025 Dec 23;25:1882. doi: 10.1186/s12885-025-15283-6 (PMC12729057; doi:10.1186/s12885-025-15283-6)
Supplement: Supplementary file 3 — Supplementary Material 3: Western Blot. [file 12885_2025_15283_MOESM3_ESM.docx]

**A**


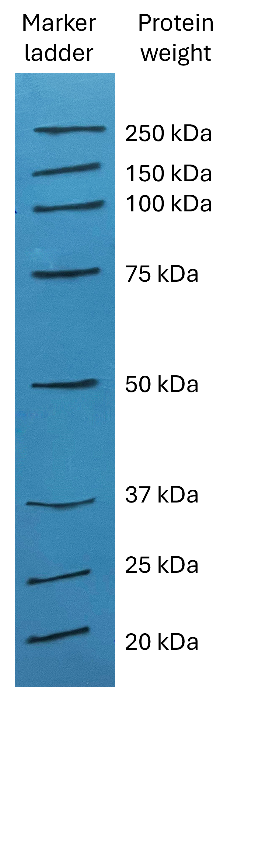

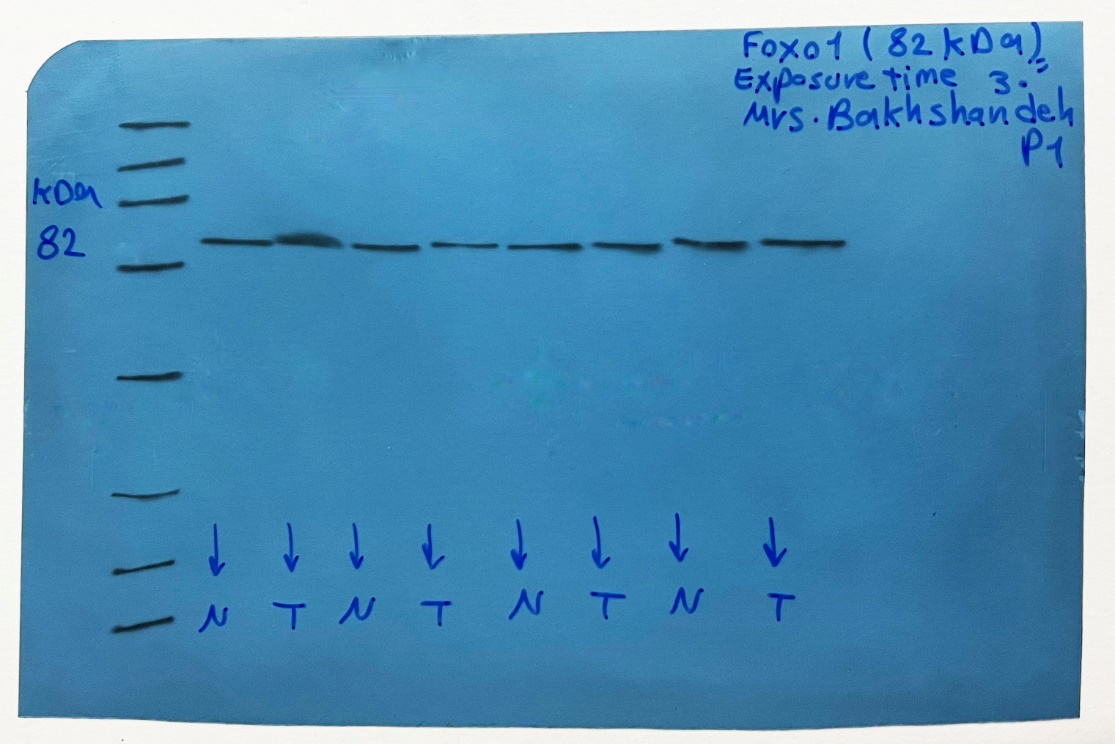


**B**


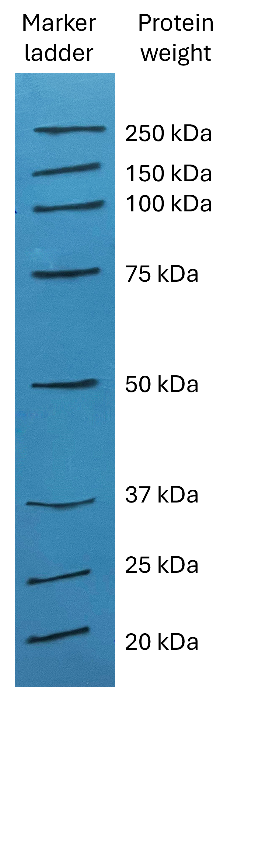

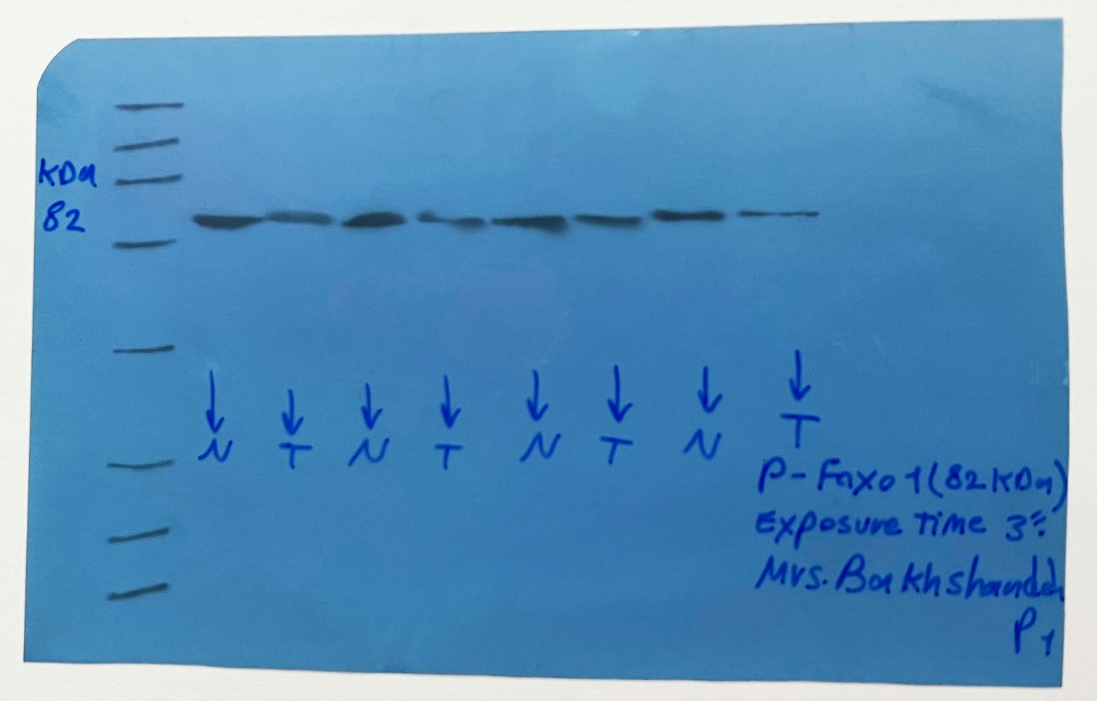


**C**


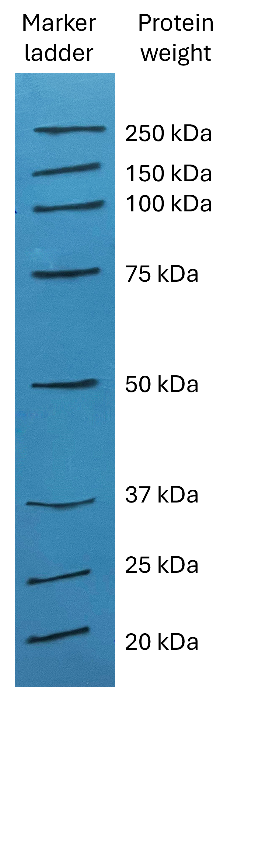

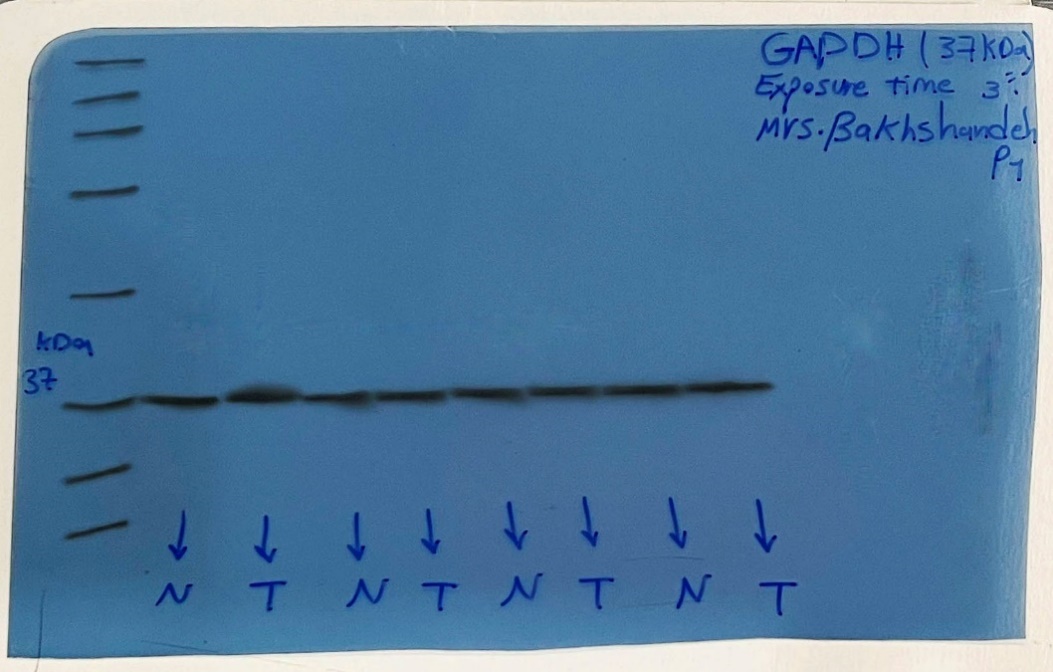


**Supplementary 3. Uncropped Western Blot images. A: FoxO1, B: phosphorylated FoxO1 (p-FoxO1, Ser256), and C: GAPDH in the laryngeal squamous cell carcinoma (LSCC).**
